# Patient-reported outcome measures of the impact of cancer on patients’ everyday lives: a systematic review

**DOI:** 10.1007/s11764-016-0580-1

**Published:** 2016-11-10

**Authors:** Susan Catt, Rachel Starkings, Valerie Shilling, Lesley Fallowfield

**Affiliations:** Sussex Health Outcomes Research and Education in Cancer (SHORE-C), Brighton and Sussex Medical School, University of Sussex, Falmer, Brighton, BN1 9RX UK

**Keywords:** Patient-reported outcomes, Cancer, Social impact, Financial burden, Quality of survival, Quality of life

## Abstract

**Purpose:**

Patients with advanced disease are living longer and commonly used patient-reported outcome measures (PROMs) may miss relevant elements of the quality of extended survival. This systematic review examines the measures used to capture aspects of the quality of survival including impact on patients’ everyday lives such as finances, work and family roles.

**Methods:**

Searches were conducted in MEDLINE, EMBASE, CINAHL and PsycINFO restricted to English language articles. Information on study characteristics, instruments and outcomes was systematically extracted and synthesised. A predefined set of criteria was used to rate the quality of studies.

**Results:**

From 2761 potentially relevant articles, 22 met all inclusion criteria, including 10 concerning financial distress, 3 on roles and responsibilities and 9 on multiple aspects of social well-being. Generally, studies were not of high quality; many lacked bias free participant selection, had confounding factors and had not accounted for all participants. High levels of financial distress were reported and were associated with multiple demographic factors such as age and income. There were few reports concerned with impacts on patients’ roles/responsibilities in everyday life although practical and emotional *s*truggles with parenting were identified. Social difficulties were common and associated with multiple factors including being a caregiver. Many studies were single time-point surveys and used non-validated measures. Exceptions were employment of the COST and Social Difficulties Inventory (SDI), validated measures of financial and social distress respectively.

**Conclusions:**

Impact on some important parts of patients’ everyday lives is insufficiently and inconsistently captured. Further PROM development focussing on roles and responsibilities, including work and caring for dependents, is warranted**.**

**Implications for Cancer Survivors:**

Factors such as finances, employment and responsibility for caring for dependants (e.g. children and elderly relatives) can affect the well-being of cancer survivors. There is a need to ensure that any instruments used to assess patients’ social well-being are broad enough to include these areas so that any difficulties arising can be better understood and appropriately supported.

## Introduction

Rapidly emerging novel treatments in oncology, particularly in advanced disease, mean that more patients are living longer [1]; for those who cannot be cured, the goal of therapy is extending survival whilst maintaining or improving quality of life. This changing landscape has implications for the tools both researchers and clinicians have at their disposal to evaluate and improve patient outcomes holistically.

The measurement of a patient’s subjective experience of both the symptoms of disease and treatment-related toxicity within clinical trials has increased [2, 3]. This practice is expanding to routine care and follow-up [4, 5] with the concept of health-related quality of life (HRQoL) subsumed under the broader term of patient-reported outcomes (PROs). Good, well-validated HRQoL instruments have been used in oncology for some time, measuring the impact of disease on functioning and well-being. Many checklists and subscales are available enabling measurement of treatment related side effects as well as symptoms of disease. These are usually designed for use with more generic HRQoL questionnaires revealing the overall impact that disease and treatment may exert on physical, functional, emotional and social well-being. The best validated, generic patient-reported outcome measures (PROMs) used most frequently are the European Organisation for Research and Treatment of Cancer (EORTC) QLQ-C30 [6] and the Functional Assessment of Cancer Therapy (FACT-G) [7] questionnaires. Both have disease-specific cancer modules or subscales, but the FACT has a wider range of treatment-specific subscales.

Although the more frequent use of PROMs is encouraging, there may well be areas of concern impacting the quality of survival that are insufficiently assessed. A previous systematic review by Muzzati and Annunziata [8] showed that some PROMs examine different and limited aspects of patients’ social well-being. For example the subscale of the EORTC QLQ-C30 refers to social and role functioning, the FACT-G to relatives and relationships and the Psychological Screen for Cancer [9] to social support. The authors of this previous systematic review [8] commented that none of the multidimensional HRQoL instruments totally captured the complexity of ‘social impact’. This led them to a systematic search of instruments dedicated solely to the assessment of the social aspects of the cancer experience (or validated in cancer populations) which yielded 27 articles relating to 14 instruments. The social dimensions covered by these tools were predominantly communication (couple/family/caregiver), relationships (with family and friends) and support (from family and friends) and are a reflection of the search terms derived from the social items listed in existing HRQoL measures and a limiting factor of the review. By deriving the search terms using this method, it is not surprising to have identified tools that measure similar issues to those of the social domains of the HRQoL instruments, but potentially covering them in greater depth. Our contention is that the literature on the social concerns and issues faced by cancer patients contains a broader spectrum of social issues than the items on those existing HRQoL instruments. Though Muzzati and Annunziata’s review systematically searched for more depth, it failed to extend its range potentially missing areas of interest. The review did identify one instrument, the Social Difficulties Inventory [10], which included an ‘everyday living’ subscale examining care of dependents, recreation and independence and a ‘money matters’ subscale with items on work, finances and welfare benefits. The social problems that patients with cancer have has been documented for some considerable time with early literature showing that problems can be experienced with employment [11–13], managing daily living [12, 14], finances [15, 16] and insurance [13, 17]. Later work has continued to reveal the social issues that cause significant concern for patients. Both qualitative research [18–20] and pure economic surveys/evaluations [21–25] illustrate that financial worry and hardship are both evident and prevalent. Furthermore, quantitative reports of return to work rates and functional abilities in terms of work performance and obstacles [26–33] and qualitative studies exploring the importance and meaning of work for patients [34, 35] serve to underline the salience of this area. Qualitative studies demonstrate that roles and responsibilities outside of work (i.e. being a parent or carer for others) are also a significant part of patients’ lives and that cancer has consequent interaction and impact on these [36–40]. Wright and colleagues [41] conducted a robust study with patients using focus groups and interviews to describe and categorise cancer patients’ social difficulties. A total of 32 social problems were identified which were able to be categorised under eight headings: (1) managing in the home, (2) health and welfare services, (3) finances, (4) employment, (5) legal matters, (6) relationships, (7) sexuality and body image and (8) recreation. This literature suggests a breadth of social aspects of patients’ lives impacted by cancer that is not traditionally captured alongside treatment toxicity and disease burden, endorsing the need for a further literature search focussed on PROs covering the areas not so well accounted for by the commonly used HRQoL measures.

The current review aims to provide summary and evaluation of PROMs used in studies for reporting the impact of cancer on wider social aspects of patients’ lives, particularly those which may be salient to living with the illness for an extended period, in other words, expanding assessments to look at lifestyle impacts under a conceptual framework which is in the early stages of delineation and referred to as ‘quality of survival’ (defined by four interconnected dimensions: survival, quality of life, side effect management and economic impact management) [42]. This will highlight what has been used, and how and where gaps may still exist which could be addressed with revision or supplementation of currently employed tools. The focus is on the patient with cancer, not caregivers nor relatives (see Shilling et al. [43] for a review of caregiver impact measures).

## Methods

### Data sources and search

We followed the general principles published by the NHS Centre for Reviews and Dissemination [44] and carried out an electronic search of databases to identify publications using PROs to evaluate the impact of cancer on social aspects of patients’ lives, specifically financial, lifestyle and occupational circumstances, along with roles and responsibilities with dependents (looking after young offspring, a spouse or elderly parents).

Searches were run in MEDLINE (MEDLINE(R) in-process and other non-indexed citations and MEDLINE(R) 1946-present) and EMBASE (1947-present) (both via OvidSP) and CINAHL (1937-present) and PsycINFO (from 1800s–present) (both via EBSCO*host*). Terms were modified as appropriate for each database and limited to English language only. A combination of controlled syntax (MeSH) and free-text terms were used. Two groups of terms were generated describing (i) the population and (ii) social aspects of interest. The terms within each group were combined with a Boolean OR command and were searched in combination using a Boolean AND command. Searches were run on 5 March 2015 and an update conducted 8 January 2016 (see Box 1 for the search strategy used for MEDLINE, adapted for other databases). The reference lists of pertinent review articles were also checked for relevant articles.

**Table:** 

Box 1: Search strategy used in MEDLINE and adjusted for other databases
1. exp neoplasm/
2. (neoplasm* or cancer).mp.
3. 1 or 2
4. (‘role burden*’ or ‘impact on leisure’ or ‘impact on lifestyle’ or ‘impact on life style’ or ‘role responsibilit*’ or ‘carer responsibilit*’ or ‘childcare role’ or ‘childcare responsibilit*’ or ‘childcare burden*’ or ‘impact on social life’ or ‘social impact’ or ‘family function’).mp.
5. exp return to work/
6. (‘return to work’ or ‘impact on occupation’).mp.
7. 5 or 6
8. (‘out of pocket’ or ‘out-of-pocket’ or ‘financ* burden*’ or ‘financ* impact’ or ‘impact on financ*’).mp.
9. 4 or 7 or 8
10. 3 and 9
11. limit 10 to english language

### Study selection criteria

Inclusion criteria were articles in the English language on any quantitative study containing the report of the impact of advanced (or secondary or metastatic or stages III or IV) cancer, as assessed by the use of PROs, on the wider social aspects of adult patients’ lives, that is on financial situation, lifestyle, occupational circumstances, roles and responsibilities with dependents (looking after young offspring or elderly parents) and there was no date restriction.

Excluded were qualitative studies, those with children or caregivers as the sole participants, those involving populations with early cancer (i.e. disease stages I or II) or the very final end-of-life stage; also excluded were studies focussed on the metrics of an instrument, reviews, editorials, letters, opinion, reports published on meeting abstracts and academic theses, any of which an English language version of the PRO had not been developed. Pure monetary costing surveys and functional workability assessment surveys were not included as these give no information on perceived impact on the patient, e.g. recording in a diary a certain amount of money was spent on a hospital parking charge does not establish if, or how, burdensome it was.

**Fig. 1:**
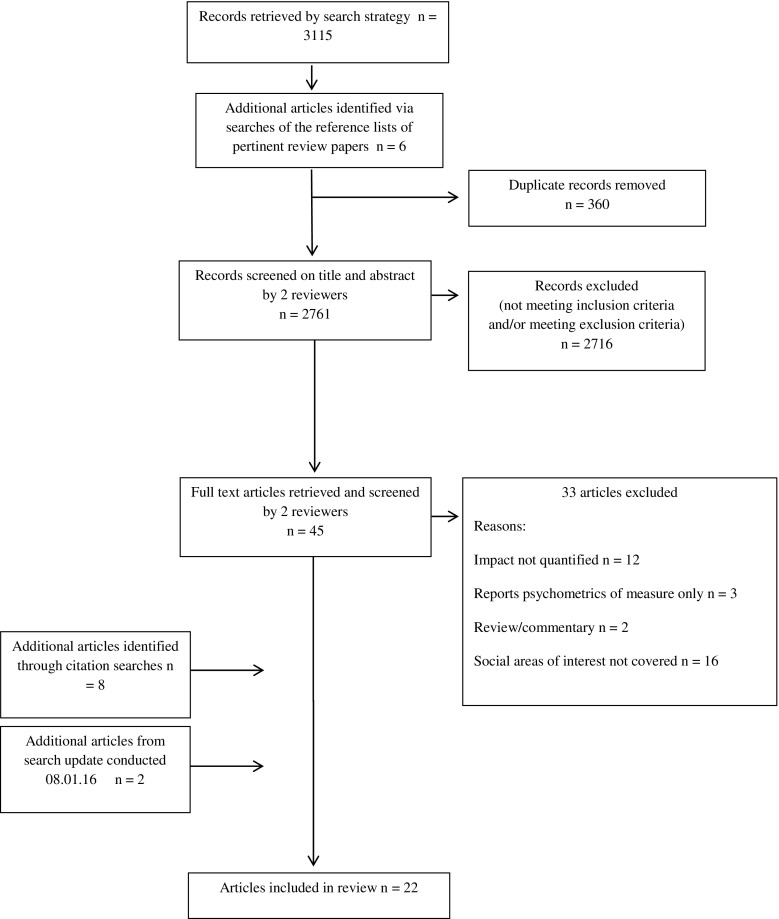
Flow chart showing identification and selection of eligible articles

A schema for identification and selection of eligible articles can be seen in Fig. 1. Titles and abstracts were screened independently by two reviewers (SC and VS) against the inclusion and exclusion criteria, and any duplicate papers were recorded and excluded. The full text of potentially relevant papers were retrieved and then further scrutinised independently by both reviewers to identify the final list included in the review. Article selection disagreements were resolved by discussion and a third reviewer (LF) provided adjudication if necessary.

### Citation chasing

Backwards citation chasing (one generation) using reference lists of all papers included in the review and forwards citation chasing (one generation) using Science Citation Index Expanded and Social Science Citation Index Expanded via Web of Science were conducted.

### Data extraction

For each included paper, the following descriptive data were extracted into a standardised form: first author name, publication year, setting/country, study design and aim, study population (cancer type and demography), number of participants, what social factor/s were assessed and how, reported outcomes and study limitations. Data were extracted and the quality of studies appraised by one reviewer (RS) and checked by a second (SC or VS). Quality appraisal criteria are shown in Box 2 and were developed in accordance with the principles published by the National Health Service Centre for Reviews and Dissemination [44]. Without evidence to inform any differential weighting amongst the criteria, none was applied. A score of one point was awarded where good evidence was provided to meet a criterion, a half point if the evidence provided was weak, and where no evidence was present a zero score was given. Higher total scores represent better quality with articles scoring 0–3.5 designated a +, scores 4–7.5 a ++ and 8–12 a +++.

**Table:** 

Box 2: Criteria for assessing the quality of studies
1. Is the aim of the study clear?
2. Is the study design/method appropriate?
3. Are the study eligibility criteria specified?
4. Is participant selection apparently bias free?
5. Is there a power calculation for sample size?
6. Is sample size adequate and response rate satisfactory? (a possible half point could be given for each)
7. Has a validated measure been used? (i.e. evidence of instrument development, content and construct validity, test-retest reliability, ability to detect change)
8. Are data analyses appropriate?
9. Are all study participants accounted for?
10. Is the study apparently free of confounding factors?
11. Are the conclusions supported by the results?
12. Are the results generalizable?

## Results

### Articles

The initial search yielded 3115 articles; an additional 6 were identified from hand searches (Fig. 1). After removal of duplicates and initial screening of titles and abstracts, 45 full texts were examined; 12 met the inclusion criteria. A further 8 articles added from the citation searches and 2 from the electronic search update resulted in 22 articles included in the review. The characteristics of the studies are summarised in Table 1, grouped under three broad subheadings dependent upon the focus of investigation: financial distress, roles and responsibilities or multiple aspects of social life. Description of the instruments used and study outcomes is summarised in Table 2.

**Table 1:** Study characteristics and quality

Author year	Design, objectives and key variables	Tumour group/s (multiple denotes >3 different sites)	Number	Age (in years)	Gender	Time since diagnosis	Quality
Financial distress							
Bennett 2009 [45]	-Single time-point survey (nested within a national study) -Changes in employment and income with comparisons between patients	Multiple	68	Inclusion—≥18 Mean—52.3 Range—22–74	69% F 31% M	6–24 months	++
Delgado-Guay 2015 [46]	-Single time-point survey -Effect of financial distress on quality of life -2 patient settings: Comprehensive Cancer Centre (CCC) versus General Public Hospital (GPH)	Multiple (advanced disease)	149	Inclusion—>18 Mean and median—60 Range—not specified	49.7% F 50.3% M	Not specified	++
Huntington 2015 [47]	-Single time-point survey -Financial toxicity (distress) is measured -Tertiary academic medical centre with 5 consultants recruiting	Multiple myeloma	100	Inclusion—not specified Mean—64.1 (s.d. 9.8) Range—38.4–90.2	53% F 47% M	Median time from diagnosis to survey completion was 31 months (IQR 14–56)	+++
Meisenberg 2015 [48]	-Single time-point survey -Patients’ views on the role of costs in relation to their treatment -Financial wellness measured	Multiple (various stages)	132	Inclusion—not specified Mean and median—61 and 64 Range—23–95	70.5% F 29.5% M	73% had initial diagnosis within the past year	++
Meneses 2011 [49]	-Longitudinal survey (baseline, 3 and 6 months) -Impact of financial burden on QoL	Breast (stages I–II)	132	Inclusion—≥21 Mean—not specified Range—21–83	100% F	≤2 years post-diagnosis and at least 1 month post-surgery, radiotherapy and/or chemotherapy	++
Pisu 2015 [50]	-Single time-point survey (within a longitudinal study) -Economic burden in racial minorities -Groups: white (W), African-American (AA) and Hispanic (H)	Colorectal (CRC) and lung (LC)	3432	Inclusion—not specified Mean—not specified Range—not specified, lower and upper ages classified as <54 and >80 for analysis purposes	CRC 45.7% F 54.3% M LC 49% F 51% M	1 year post-diagnosis	+
Regenbogen 2014 [51]	-Single time-point survey -Relationship between (self-reported) perioperative complications and financial burden	Colorectal (stage III)	937	Inclusion—≥21 Mean—not specified Range—not specified, grouped as <50, 50–64, 65–74 and ≥75	46% F 53% M (missing data apparent)	3–12 months post-resection	++
Rogers 2011 [52]	-Single time-point survey -Relationship between Health related QoL and financial burden	Head and neck primary SCC	447	Inclusion—upper age limit 85 Mean—not specified Range—not specified, grouped as <55, 55–64, 65–74 and 75–84	72% M 28% F	≤12–60+ months	++
Sharp 2013 [53]	-Single time-point survey -Relationship between cancer related financial stress (objective) and strain (subjective) and psychological well-being	Breast, prostate and lung	654	Inclusion—not specified Mean—not specified (median 57) Range—26–88	69% F 31% M (5 missing responses noted)	Not specified	++
Zafar 2013 [54]	-Single time-point survey (data from out-of-pocket expense diaries not included in review) -Impact from associated healthcare costs on the well-being and treatment of insured patients	Multiple (solid tumours)	254	Inclusion—not specified Mean (and median)—64 Range—29–88	86% F 14% M	Not specified (stated patients were undergoing active chemotherapy or hormone therapy)	+
Roles and responsibilities							
Moore 2015 [55]	-Single time-point survey -Model connecting parental cancer to child distress based on the parent’s perception	Multiple	194	Inclusion—not specified (adult) Mean—not specified (median 46) Range—26–65	73% F 27% M	Range from 2 to 237 months (median 14 months)	++
Park 2015 [56]	-Single time-point survey -Relationship between parenting concerns, quality of life, and depression and anxiety in patients with advanced disease with dependent children	Multiple (stage IV solid tumour groups)	63	Inclusion—≥18 Mean—43.6 (8.2) Range—21–63	68.3% F 31.7% M	Mean: 17 months (s.d. 18) since metastatic diagnosis	++
Walsh 2005 [57]	-Single time-point survey -Patient perspective on their role changes as wife and mother	Breast	204	Inclusion—≤50 Mean—43.45 (s.d. 6.20) Range—25–50	100% F	3–36 months post-first diagnosis	+++
Multiple aspects of social life							
De Groot 2005 [58]	-Single time-point survey -Impact of psychosocial concerns on both patients and partners	Cervical	26 dyads	Inclusion—not specified Mean Females—44.35 (s.d. 11.1) Males—47.4 (s.d. 12.2) Range—not specified	50% F 50% M	Pre- to 2 years post-treatment	++
Devins 2006 [59]	-Single time-point survey -Impact of modifying variables (age, income, education and recent stressful life events) on the degree of illness intrusiveness	Multiple (NB same data set as Mah 2011)	656	Inclusion—not specified Mean—57.8 (s.d. 13.9) Range—not specified	49% F 51% M	Mean: 5 years post-treatment (newly diagnosed patients were excluded)	++
Mah 2011 [60]	-Single time-point survey -Review of illness intrusiveness across tumour groups	Multiple (NB same data set as Devins 2006)	656	Inclusion—not specified Mean Prostate—66.99 (s.d. 10.45) Breast—56.71 (s.d. 12.27) Lymphoma—52.39 (s.d. 14.86) H&N—50.97 (s.d. 13.77) GI—56.56 (s.d. 11.85) Lung—62.08 (s.d. 11.55) Range—not specified	49% F 51% M	Tumour group means provided in years: Prostate—5.30 (s.d. 5.81) Breast—6.11 (s.d. 6.16) Lymphoma—5.51 (s.d. 5.88) H&N—4.01 (s.d. 5.87) GI—3.08 (s.d. 5.38) Lung—4.03 (s.d. 5.05)	++
Paul 2013 [61]	-Single time-point survey -Barriers to accessing care in metropolitan and non-metropolitan areas -Financial and social impacts of the disease	Haematological	268	Inclusion—18–80 Mean—59.5 (s.d. 13.4) Range—not specified	41% F 59% M (missing data declared by authors and only females are analysed in this paper)	Up to 3 years post-diagnosis	+
Schimmer 2001 [62]	-Single time-point survey -Impact of illness intrusiveness on well-being -Comparison between autologous blood and marrow transplantation (ABMT) and solid organ transplant patients	ABMT patients and solid organ transplant data from a previous paper	ABMT 44 Kidney 357 Liver 150 Lung 77 Heart 60	Inclusion—not specified Mean ABMT mean—46 (s.d. 10.9) Transplant means: Kidney—50.8 (s.d. 12.0) Liver—49.7 (s.d. 12.0) Lung—45.9 (s.d. 12.0) Heart—52.2 (s.d. 10.0) Range—not specified	ABMT 39% F 61% M Kidney 47% F 53% M Liver 54% F 46% M Lung 45% F 55% M Heart 13% F 87% M	Years since transplant: ABMT—4.6 (s.d. 2.8) Kidney—5.0 (s.d. 2.6) Liver—2.6 (s.d. 1.9) Lung—2.2 (s.d. 1.7) Heart—3.2 (s.d. 2.1)	+
Simon 2008 [63]	-Longitudinal survey (~2 and 10 months post-diagnosis) -Relationship between socioeconomic status and psychosocial outcomes	Breast, prostate and colorectal	352	Inclusion—not specified Mean—not specified Range—29–89 (52% >65)	68% F 32% M	1–3 months post-diagnosis	++
Sohl 2014 [64]	-Longitudinal survey (baseline, 6, 12 and 18 months) -Examining patterns of illness intrusiveness over time	Breast (stages I, II, III)	653	Inclusion—≥25 Mean—54.9 (s.d. 12.6) Range—not specified	100% F	≤8 months post-diagnosis	++
Wright 2005 [65]	-Data pulled from 3 studies: -Study 1 (psychometric evaluation of Social Difficulties Inventory (SDI), including test-retest) -Study 2 (cross-sectional, single time-point survey) -Study 3 (longitudinal survey, assessments at baseline, 6-, 12- and 24-month examining the relationship between social difficulties and psychological distress with deprivation)	Multiple	609 = (270 + 189 + 150)	Inclusion—not specified Mean—not specified Range—not specified, grouped as ≤40, 41–60 and ≥61	50% F 50% M	A range across the 3 studies (N.B. <3 months in study 3)	+++
Wright 2015 [66]	-Single time-point survey -Examining social difficulties, impact on social aspects of life including home and work, finances and relationships and recreation	Colorectal	21,802 SDI-16—17,830/21,802 SDI-21—16,962/21,802	Inclusion—>16 Mean—not specified Range—not specified, grouped as <55, 55–64, 65–74, 75–84 and ≥85	All respondents 41.8% F 58.2% M SD-16 39.9% F 60.1% M	12–36 months post-diagnosis	+++

*s.d.* standard deviation

*+* score between 0 and 3.5 on quality criteria, *++* score between 4 and 7.5, *+++* score between 8 and 12

**Table 2:** Instruments and study outcomes

Instrument and studies	Instrument description	Main findings
Financial distress		
Work, Life and Finances subscale (study-specific instrument) Bennett 2009 [45]	Questions: -25 covering employment, household income, health insurance -11 about changes made to work circumstances (e.g. reduced working hours) within first 6 months after treatment completion Rating scale: 3-point ‘not at all’, ‘a little’, ‘a lot’	-35 respondents said that they were the main earner in their household before diagnosis, 26 maintained this after treatment -Of the 9 who said that they were no longer the main earner, 5 said that this was due to cancer -37% reported having a reduced household income, and all said that this was due to cancer -72% reported utilising at least one method of economising -No one reported going without treatment but 62% reduced or stopped trips to the shops
Financial Questionnaire (author generated) Delgado-Guay 2015 [46]	Questions: -4 statements about impact of financial distress on ‘physical’, ‘social’, ‘spiritual’ and ‘emotional’ well-being -4 terms, ‘subjective financial burden’, ‘financial concerns’, ‘financial difficulties’, ‘financial worries’ Rating scales: Statements were scored on 5-point scale ranging from ‘strongly agree’ to ‘strongly disagree’ Terms were scored on a 11-point scale with 0 as ‘absent’ and 10 as the ‘worst possible’	-Educational levels differed between the Comprehensive Cancer Centre (CCC) and the General Public Hospital (GPH) patients (58 and 19% respectively had college education or an advanced degree, *p* < 0.0001), as did monthly income (median of $3000 versus $940, *p* = 0.0017) -The distribution of race/ethnicity also differed (*p* < 0.0001) (62 versus 18% were white at CCC and GPH respectively, 27 versus 38% were black and 9 versus 38% were Hispanic). -Financial distress (FD) was highly prevalent, as reported by 86 and 90% of patients from the CCC and GPH respectively -Median FD scores for the GPH patients were 4 (IQR 1–7) versus 8 (IQR 3–10) for the CCC patients (*p* = 0.0003) -30% of patients had more FD than physical distress, and there was no significant group difference -31% had more FD than distress from physical functioning, more so for the GPH group (39 versus 23% with *p* = 0.051) -43% had more FD than social/family distress, more so for the GPH group (54 versus 33% with *p* = 0.0085) -37% had more FD than emotional distress, more so for the GPH group (46 versus 29% with *p* = 0.041)
The COST measure Huntington 2015 [47]	Questions: -11-item PRO developed for patients with advanced malignancies covering: ‘having enough money for treatment costs’, ‘expectation about out-of-pocket medical expenses’, ‘worry about future financial problems’, ‘perceived control over money spent on care’, ‘frustration at inability to work/contribute’, ‘satisfaction with current finances’, ‘ability to meet monthly expenses’, ‘feeling financially stressed’, ‘concern about maintaining job and income’, ‘cancer or treatment reducing financial satisfaction’, ‘feeling in control of finances’ -1 additional question asking to rate self-reported level of financial burden -1 additional question asking to rate expectation about treatment costs -list of coping strategies that patients indicate which if any they employ Rating scales: -The COST measure has a 5-point scale 0 = ‘not at all’, 1 = ‘a little bit’, 2 = ‘somewhat’, 3 = ‘quite a bit’, 4 = ‘very much’ (scores are totalled and range between 0 and 44 with lower scores indicative of greater burden) -Self-reported level of financial burden rated on a 4-point scale ‘not at all’, ‘minor’, ‘moderate’, ‘significant’ -Expectation about treatments costs rated on 3-point scale ‘lower than expected’, ‘as expected’, ‘higher than expected’	-Mean COST score was 23 (s.d. 11.1), and the median (23.5) was used to stratify higher financial burden (COST score ≤ 23) versus lower (score > 23) -71% reported at least minor financial burden -59% of indicated treatment costs were higher than expected, more so for those with higher financial burden than lower (76 versus 42% respectively, *p* = 0.00057) -46% used savings to pay for treatment, 21% borrowed money to cover treatment costs and 17% reported treatment delays due to cost -36% applied for financial assistance; this did not differ significantly between higher and lower financial burden groups -Patients with higher financial burden were more likely to employ general financial coping strategies, e.g. reduce spending on basic goods and leisure activities, use savings for care, borrow money to pay for treatment (all *p*s < 0.0001) -Patients with higher financial burden were more likely to employ treatment-related financial coping strategies, e.g. delay treatment initiation (*p* = 0.0030), fill only part of a myeloma therapy prescription (*p* = 0.0077), stop myeloma therapy (*p* = 0.0011), skip a clinic visit to save on costs (*p* = 0.027) -Younger age (*p* = 0.00092), non-married status (*p* = 0.0074), longer time since diagnosis (*p* = 0.042) and lower household income (*p* = 0.0031) were associated with higher financial burden on the COST measure in multivariate analysis
Personal Financial Wellness (PFW) Scale -formally known as ‘The Incharge Financial Distress/Financial Well-Being Scale’ Meisenberg 2015 [48]	Questions: -8-item PRO that assesses financial distress covering ‘level of financial stress today’, ‘satisfaction with present financial situation’, ‘level of comfort with current financial situation’, ‘frequency of foregoing a social activity, e.g. eating out, seeing a movie’, ‘frequency of having to live paycheck to paycheck’, ‘frequency of worry about meeting normal monthly living expenses’, ‘confidence in ability to pay for a financial emergency’, ‘general level of stress due to finances’ -1 additional dichotomous item asked ‘have you changed your spending habits, e.g. reduced leisure spending due to care costs?’ -1 additional item asked to what extent do you agree with the statement ‘I believe that being sick has or will hurt me financially’ Rating scales: -Each item on the PFW is rated on a 10-point scale of 1–10 (some items are reverse scored) -Lower scores on the PFW indicate greater financial distress and recommended cutoffs provide categories of ‘high distress’ (scores <4.4), ‘average distress’ (scores 4.5–6.4), ‘low distress’ (scores >6.5) -The item on financial hurt was rated on a 5-point scale from 1 = ‘strongly disagree’ to 5 = ‘strongly agree’	-Mean financial distress score was 5.11 (s.d. 2.77) compared to a general population mean of 5.7 (lower score indicating greater distress) -47% of participants reported high levels of financial distress -50.4% of participants agreed or strongly agreed that being sick had hurt them financially -52.3% had changed their spending habits as a result of treatment costs -6.1% had reduced medication adherence due to costs -25.8% had increased their debt, 22% had lapsed on bills and 1.5% had declared bankruptcy -Those with higher income were less likely to have endorsed experiencing financial impact from treatment (*p* < 0.001, CI −0.53 to −0.18) -Marital status, medical insurance status, treatment and age were not associated with the perception that treatment had resulted in a financial impact
Breast Cancer Finances Survey (adapted from Given 1994 [67]) Meneses 2012 [49]	Questions: -4 items about changes in work( work motivation, productivity, quality and missed work days) -15 items about financial hardship (i.e. income reduction, sold house, used up savings, spouse lost wages due to caring duties, etc.) Rating scale: each item was endorsed with a binary ‘yes’/’no’ response	-Over 50% of participants reported at least one economic burden event related to either their work or a financial hardship within 6 months following treatment completion -Items most endorsed at baseline were productivity at work (27%), missing days (38%), sacrificed leisure, e.g. holidays (40%), income reduction (35%) and used savings (27%) -Participants reported a mean of 2.94 burden items at baseline, 2.45 at month 3 and 2.25 at month 6 -The endorsement of 5 items significantly decreased from baseline to 6 months: reduced work motivation (23 to 12%, *p* = 0.016), productivity (27 to 12%, *p* = 0.002), quality (17 to 7%, *p* = 0.01), missing days (38 to 19%, *p* < 0.001) and sacrificed leisure, e.g. holidays (40 to 31%, *p* = 0.001) -The item, ‘increase in health insurance premiums’, was increasingly endorsed across the 6 months (7 to 16%, *p* = 0.022)
Financial Questionnaire (author generated) Pisu 2015 [50]	Questions: -1 item about current level of financial difficulty: ‘how difficult is it to live on the household income right now?’ -2 about anticipated financial difficulty: ‘how much will family experience financial hardship in next 2 months?’ and ‘how much will standards of living have to be reduced in next 2 months?’ Rating scales: -Current financial difficulty scored on a 5-point scale (‘not at all’, ‘somewhat’, ‘difficult’, ‘very’ and ‘extremely’) -Anticipated financial difficulty items scored on a 4-point scale (‘not at all’, ‘a little’, ‘moderately’, ‘a great deal’) -For the purposes of analysis all response scores were dichotomised with 0 for ‘not at all’ and 1 for all other scores beyond ‘not at all’	-In both the lung cancer (LC) and the colorectal cancer (CC) cohorts, patients of minority race/ethnic background had lower incomes (*p* < 0.05) and were less likely to have prescription drug coverage (*p* < 0.05) -Across all participants, ≥40% reported financial hardship (defined as having current difficulty with household income or anticipating financial hardship or anticipating having to reduce standards of living) -Minority races reported greater financial hardship in both the LC (white 50%, African-American 69%, Hispanic 59%, *p* < 0.05) and CC (white 41%, African-American 67%, Hispanic 59%, *p* < 0.05) cohorts -In statistical modelling, lack of prescription drug coverage and lower income were associated with financial hardship and it was suggested could explain to some extent the racial/ethnic disparities
Financial Questionnaire (author generated) Regenbogen 2014 [51]	Questions: -7 items covered financial adjustments made due to disease (‘used savings’, ‘borrowed money’, ‘couldn’t pay bills/credit cards’, ‘spent less on food/clothing’, ‘less spent on other family health’, ‘spent less on leisure’, ‘generally spent less’ -1 item about financial worry (‘how much do you worry about financial problems resulting from your cancer/treatments?’) Rating scales: -The 7 financial items required a binary response ‘yes’ or ‘no’ and these items were summed to give a composite measure of financial burden (range 0–7) -Financial worry was scored on a 5-point scale 1 = ‘not at all’ to 5 = ‘very much’ (scores of 1–3 were considered ‘low worry’ and scores of 4–5 ‘high worry’)	-38% reported making no financial adjustments on the composite burden measure, 29% endorsed 1–2 items, 22% 3–4 items and 11% 5–7 items -Higher financial burden scores were significantly associated with higher financial worry (*p* < 0.001) -Patients with self-reported post-operative complications were more likely to endorse items of financial burden (70 versus 59% endorsed 1 or more burden items, *p* < 0.001); NB 24% of patients said they had complications -Those with complications reported higher levels of financial worry (61 versus 52%, *p* = 0.01) -Multiple regression showed that location (metropolitan versus others), age, income, chemotherapy and health status were all related independently to financial burden -The relationship between financial burden and complications remained after controlling for the above covariates plus sex, race, marital status, education and co-morbidities (crude scores 2.15 versus 1.66, *p* = 0.03; adjusted scores 2.21 versus 1.69, *p* < 0.001)
Cost of Head and Neck Cancer Questionnaire (author generated following review of SDI-21 and EORTC-QLQ-C30 instruments) Rogers 2011 [52]	Questions: -1 item asking if working status had been affected by cancer -17 items covering common life expenses (e.g. food, heating, travel, childcare, mortgage, clothes) -1 question to nominate the 3 life expenses, from item list of 17 common life expenses, most impacted by cancer and indicates if statutory financial help had been sought and received -5 items scored for difficulty experienced within the past month: work or if student education, planning for the future, living environment (e.g. housing conditions), benefits (e.g. sick pay) and financial services (e.g. mortgage) -1 item on satisfaction with coping with own finances -1 item about financial difficulty in the past week Rating scales: -5-point burden scale ‘none’, ‘little’, ‘moderate’, ‘large’ or ‘not applicable’ for rating common life expenses -4-point difficulty in the past month scale ‘no difficulty’, ‘a little’, ‘quite a bit’, ‘very much’ -6-point satisfaction with financial coping scale ‘very satisfied’ to ‘very unsatisfied’ -4-point financial difficulty scale ‘not at all’, ‘a little’, ‘quite a bit’, ‘very much’	-31% of respondents said cancer had affected their working status -54% of respondents had at least one moderate or large burden and 17% had at least 5 -More patients under 65 years indicated at least 3 financial issues that were moderate or large burdens (48% of 55 years old, 44% of 55–64 years old, 21% of 65–84 years old, *p* < 0.001) -The most notable financial costs that were a moderate or large burden to patients were expense on petrol (25%) and home heating (24%) -39% of patients applied for state financial assistance, as a result of cancer, with 71% of these receiving help -25% were moderately or very dissatisfied about coping with their finances -Those with worse physical and social-emotional functioning experienced more financial burden, difficult life circumstances in the past month, financial difficulty and loss of earnings in the past week were more dissatisfied with their financial coping and sought more state financial help
Financial Measure (author generated) Sharp 2013 [53]	Questions: -1 question assessed the patient’s perception of the impact of the cancer diagnosis on the household’s ability to make ends meet, i.e. difficulty of making ends meet (labelled ‘financial stress’) -1 question assessed how concerned the patient felt about their household’s financial situation since their cancer diagnosis (labelled ‘financial strain’) Rating scale: 7-point scale ‘much more difficult/very concerned’ to ‘much less difficult/less concerned’	-49% reported increased financial stress and 32% increased financial strain due to the cancer -Depression was raised twofold to threefold in those reporting increased cancer-related financial stress (OR = 2.79, 95% CI 1.87–4.17) and strain (OR = 3.56, 95% CI 2.23–5.67) -Anxiety was raised threefold and greater in those reporting increased cancer-related financial stress (OR = 3.44, 95% CI 2.21–5.35) and strain (OR = 4.43, 95% CI 2.65–7.39)
Financial Questionnaire (author generated) Zafar 2013 [54]	Questions: -1 subjective financial burden question ‘how much financial burden has resulted from cancer-related out-of-pocket expenses?’ -7 descriptions of ways to alter obtaining prescription medication to reduce costs, e.g. shop at cheapest pharmacy -3 descriptors of lifestyle changes to assist coping with financial impact of prescription medications, e.g. spend less on food -3 descriptors of ways to alter medication use to reduce costs, e.g. take less than prescribed amount -5 descriptors of ways to alter care to reduce costs, e.g. cancelling clinic appointment -7 descriptors of lifestyle changes to assist coping with financial impact of care services, e.g. sold possessions Rating scales: -5-point financial burden scale ‘not at all’, ‘minor’, ‘moderate’ ‘significant’, ‘catastrophic’ -For all coping strategy descriptors, a binary response ‘used’ or ‘not used’	-42% reported a significant or catastrophic subjective financial burden -To reduce medication costs 55% obtained samples from doctor, 48% asked for a cheaper drug to be prescribed, 47% shopped around for lowest drug prices and 19% took less than prescribed amount -Most frequent lifestyle changes made to save money were reduced leisure activities (68%), cut-backs on food and clothing (46%), using savings (46%) and borrowing money/using credit (35%) -Higher subjective financial burden was associated with application for statutory financial help (*p* = 0.007) and talking to the doctor about care costs (*p* = 0.02) -Lower subjective financial burden was associated with age ≥65 years (*p* < 0.001) and smaller household size (*p* = 0.008)
Roles and responsibilities		
Parenting Concerns Questionnaire (PCQ) Moore 2015 [55] (N.B. this paper also used the Parental Efficacy Beliefs Scale, see below) Park 2015 [56]	Questions: -Practical Impact subscale of 5 items about concerns relating to the practical impact of the parent’s illness on the child -Emotional Impact subscale of 5 items about concerns relating to the emotional impact of the parent’s illness on the child -Concerns about co-parent subscale of 5 items about concerns relating to co-parent’s abilities to perform their role Rating scale: -5-point scale 1 = ‘not at all concerned’ to 5 = ‘extremely concerned’ -ratings from all 15 items can be summed for a total score, and scores for the 3 subscales can also be calculated and reported	Moore 2015 -Higher PCQ total score and greater concerns on the practical and emotional subscales were all associated with decline in parental efficacy (as rated using Parental Efficacy Beliefs Scale below)—all *p*s < 0.001, but not the co-parent concerns scale -Decline in patients’ perceptions of co-parent efficacy was also associated with total PCQ score (*p* < 0.001), practical impact scale (*p* < 0.01), and co-parent concerns scale (*p* < 0.01), but not the emotional impact scale Park 2015 -Mean total score was 2.3 (s.d. 0.9) reflecting ‘mild’ to ‘moderate’ concerns -Means for the subscales were practical subscale 2.5 (s.d. 1.1), emotional subscale 2.5 (s.d. 1.1) and co-parent subscale 1.8 (1.0) -Higher PCQ scores were associated with single marital status (*p* = 0.05), poor functional status (*p* < 0.001), poorer quality of life (*p* < 0.001), anxiety (*p* < 0.001), depression (*p* < 0.002) and lack of social support (*p* < 0.002) -The highest scoring worries patients had were ‘how their child would cope with their death’ (mean 4.0, s.d. 1.2), ‘current emotional impact of their illness on their child (mean 3.3, s.d. 1.2) and the emotional impact of the disease on their partner (mean 3.1, s.d. 1.3)
Parental Efficacy Beliefs Scale (author generated) Moore 2015 [55]	Questions: -1 item asking the patient ‘how well were you able to meet your children’s needs before diagnosis?’ -1 item asking ‘how well are you now able to meet the needs of your children now?’ -2 further items rate the patient perceptions of the co-parent meeting the needs of the children before and after diagnosis Rating scale: 5-point scale (‘not well at all’, ‘not that well’, ‘well enough’, ‘very well’, ‘extremely well’)	-60% of patients felt that they had met their children’s needs ‘extremely well’ before diagnosis, but only 11% felt this way after diagnosis -42% of patients felt their co-parent had met their children’s needs ‘extremely well’ before diagnosis; 26% felt so afterwards -Mean scores for patients’ parenting efficacy dropped significantly after diagnosis (before = 4.53 (s.d. 0.63) versus after = 3.4 (s.d. 0.88), *p* < 0.0001); for co-parent, efficacy rating dropped from 3.89 (s.d. 1.20) to 3.57 (s.d. 1.28), *p* < 0.0001 -Mean parental efficacy change score was −1.13 with no significant difference between mothers and fathers. For co-parents, this was −0.32 and fathers perceived a bigger decline in their partner’s ability after diagnosis than mothers did -Decline in parental efficacy was associated with more frequent medical clinic visits (*p* = 0.001), poorer health-related quality of life (*p* < 0.001) and depression (*p* < 0.001)
CARES Marital and Relationship with Children subscale Walsh 2005 [57] (adapted the time frame so that patients were asked to respond reflecting on the time since diagnosis rather than ‘in the past month’ which is standard for administration)	Questions: -18-item ‘Marital subscale’ about woman’s relationship with her spouse/partner covering 5 domains (communication, affection, interaction, partner neglect and overprotection) -3-item ‘relationships with children subscale’ about communication with and taking care of children Rating scale: 5-point scale (0 = ‘not at all’ to 4 = ‘very much’) with higher scores indicative of greater problems	-On the Marital subscale, the communication domain had the highest mean (1.26, max score 4) score indicating the most problematic area -The most frequently endorsed items for being a problem within spouse/partner communication were ‘talking about what may happen after the patient’s death’ (52.8%) and ‘talking about cancer’ (37.4%) -On the Marital subscale the other domains of affection, intimacy, neglect and partner overprotection were not reported as problematic by many women -The overall mean for the relationships with children was low (0.69, max score 4), but percentages of women indicating a problem (scoring ‘fair amount’, ‘much’ or ‘very much’) on the individual items showed that 19.7% had difficulty with ‘helping their children talk about their cancer’, 16.8% ‘helping their children cope’ and 12.7% ‘taking care of their children as a result of having breast cancer’
Multiple aspects of social life		
Illness intrusiveness ratings scale (IIRS) De Groot 2005 [58] Devins 2006 [59] and Mah 2011 [60] (N.B. these papers report different analyses of the same data set) Schimmer 2001 [62] Sohl 2014 [64]	Questions: -13-item scale measuring degree to which a person’s illness and/or treatment interferes with 3 life domains -Instrumental domain (4 items): health, work, active recreation, e.g. sport, financial situation -Intimacy domain (2 items): sex life, relationships with spouse/partner -Relationships/personal domain (6 items): passive recreation, e.g. reading, or listening to music, family relationships, other social relationships, self-expression/improvement, community and civic involvement, religious expression -1 independent item about diet Rating scale: 7-point (1 = ‘not very much’ to 7 = ‘very much’)	De Groot 2005 -Illness intrusiveness was greater for those with advanced disease than early stage (mean 3.6 concerns, 95% CI = 2.8–4.5 versus mean 2.5 concerns, 95% CI = 1.8–3.2, *p* < 0.049) -Most frequently reported concerns were prognosis and intimacy (sex life and spousal relationship) -Illness intrusiveness associated with treatment being more recent where treatment completion <12 months ago was associated with more concerns than if treatment was completed >12 months ago (mean 3.5 concerns, 95% CI = 2.8–4.2 versus mean 2.2 concerns, 95% CI = 1.3–3.1, *p* < 0.049) -The illness intrusiveness reduction associated with greater time since treatment completion showed an interaction that was followed up by post-hoc Tukey’s B tests. This indicated the significant reductions occurred in only 3 areas: communication with the treatment team, relationship with spouse/partner, relationship with others Devins 2006 -Higher rates of illness intrusiveness were reported by patients who were younger (*p* < 0.014), on a lower income (*p* < 0.002) and who had experienced one or more stressful life events (*p* < 0.014) -On average illness intrusiveness was reported as highest for the instrumental domain (3.4, CI = 3.2–3.5), followed by intimacy (2.8, CI = 2.6–3.0) and then relationships/personal (2.2, CI = 2.1–2.3) -There was a main effect of cancer type modifying levels of intrusiveness (*p* < 0.004) with highest scores for gastrointestinal (mean = 3.4) and lowest for prostate (mean = 2.2) -There was also a significant interaction between type of cancer and illness intrusiveness domain (*p* < 0.0005). All cancer types except prostate reported illness intrusiveness highest for the instrumental domain, followed by intimacy and then relationships/personal -For patients with prostate cancer illness intrusiveness was highest for the intimacy domain, followed by instrumental, and then relationships/personal Mah 2011 -Women, regardless of type of cancer, reported highest illness intrusiveness for the instrumental domain (mean = 3.39), followed by intimacy (2.49), then relationships/personal (2.27). The type of cancer had no interactive effect and all of the differences between domains were significant (all *p*s ≤ 0.03) -Men with prostate cancer reported significantly lower total illness intrusiveness than men with either gastrointestinal (36.63 versus 28.56, *p* = 0.01) or lung cancer (39.00 versus 28.56, *p* = 0.001). No other significant cancer type group differences were evident for total scores Schimmer 2001 -Mean total illness intrusiveness score = 37.2 (s.d. 17.6) for patients with blood/bone marrow transplantation -Higher illness intrusiveness was associated with receiving transplantation more recently (*p* = 0.01), with depression (*r* = 0.48, *p* < 0.0001), and hopelessness (*r* = 0.58, *p* < 0.0001); lower levels with positive affect (*r* = −0.54, *p* < 0.0001) and happiness (*r* = −0.44, *p* = 0.004) -No significant correlations between illness intrusiveness and age, gender, education, underlying diagnosis (myeloma, leukaemia, Hodgkin disease or breast cancer), or employment Sohl 2014 -92% had early stage disease -The majority (52–65%) of these women with breast cancer reported low levels of illness intrusiveness in all three domains during the 2 years since treatment -The instrumental domain showed most (52%) had constantly low scores, 34% started high and decreased over time and 14% had scores that remained high throughout -The intimacy domain showed most (60%) had constantly low scores, 30% started high and decreased over time and 10% had scores that remained high throughout -The relationship/personal domain showed that most (65%) had constantly low scores, 9% started high and decreased over time, 9% started low and increased over time, 10% had high scores throughout -Being older, not having children <18 years at home, having stage 1 disease, having fewer symptoms and better psychosocial well-being were all associated with constantly low illness intrusiveness (all *p*s < 0.05)
Social and Financial Impacts Questionnaire (author generated) Paul 2013 [61]	Questions: -5 items related to social impact: ‘had reduced access to children’, ‘needed help to care for family’, ‘missed family events/children’s activities’, ‘missed important social or religious activities’, ‘lost contact with friends’ -6 items related to financial impact: ‘time-off work’, ‘income loss’, ‘job loss’, ‘bills payment problems’, ‘daily expenses problems’, ‘used up savings’ -9 items related to personal expenses incurred: ‘hospital travel’, ‘hospital accommodation’, ‘hospital parking’, ‘drugs/treatments’, ‘other medical supplies’, ‘own homecare’, ‘childcare’, ‘other dependant’s care’, gardening/housework’ -8 items related to things that could reduce social and financial impacts: ‘local treatment’, ‘out-of-hours clinic times’, ‘weekend clinics’, ‘free hospital parking’, ‘free hospital transport’, ‘free medications/treatments, ‘direct financial support’, ‘none’ Rating scale: on each of the 3 lists, participants select all those items that are relevant	-64% of participants reported at least one cancer-related financial or social impact in their lives; most frequently reported were time-off work (44%), reduced income (31%), missing family events (23%) and difficulty paying bills (21%) -Metropolitan respondents reported significantly more financial impact than their non-metropolitan counterparts (*p* = 0.014), but no similar difference was evident for social impact -Individuals at a higher risk of experiencing financial or social impacts were younger, employed, stressed and reported a personal expense -45% said a cancer related out-of-pocket expense had been incurred in the past month including paying for: hospital/clinic parking (33%), travel to medical appointments (30%), treatment drugs (24%), home help or gardener (8%) -58% reported at least one item that would (was it to be available) have reduced the social and financial impacts of the disease on their daily life; most frequently endorsed were free hospital parking (37%), free medications/treatments (29%) and access to treatment locally (20%)
Social Difficulties Inventory (SDI-21) Simon 2008 [53] Wright 2005 [65] (provides an overview of data from three separate studies) Wright 2015 [66] (only included participants who had completed all of the SD-16 but did report on the full SDI-21 for these individuals)	Questions: -21 items covering a range of everyday difficulties commonly encountered by patients with cancer. The inventory is subdivided into: -Everyday living subscale (6 items) about: independence, domestic chores, personal care, care of dependents, getting around, recreation -Money matters subscale (5 items) about: welfare benefits, finances, financial services, work, planning the future -Self and other subscale (5 items) about: support for close relatives, communication with close relatives, communication with others, body image, isolation -5 standalone items about: sex, plans to have a family, discrimination due to the illness, living conditions, holidays Rating scale: -4-point scale rating difficulty from 0 = ‘no’ to 3 = ‘very much’ -A summary score of the 16 items on the 3 subscales can be reported (SD-16) -3 subscales can be individually reported	Simon 2008 -Having invasive disease (*p* < 0.001), surgery (*p* < 0.01) and chemotherapy (*p* < 0.001) were associated with reporting more social difficulties -Within 2 months of diagnosis, those with lower socioeconomic status (SES) had more social difficulties (mean 10.2 versus 8.0, *p* < 0.01), and this difference persisted after controlling for age, gender, disease site and stage and treatments received -A significant decrease in social difficulties was shown at 10 months follow-up (mean 7.9 versus 5.5, *p* < 0.001), and the apparent disparity between low and high SES had disappeared by follow-up as there was no interaction for SES Wright 2005 -The authors reported that cancer and cancer treatments at all stages had an impact on social aspects of patient’s lives with 275/609 (45%) reporting high levels (summary score ≥ 9) of difficulty -Stage of disease (*p* < 0.0001), age (*p* < 0.009) and deprivation (*p* < 0.048) significantly influenced prevalence of social difficulties (younger patients, those with more advanced disease and, to a lesser extent, those from more deprived areas reported more difficulties) -An interaction between stage of disease, deprivation, age and sex was found (*p* < 0.015); post-hoc analysis on this interaction showed that significantly more social difficulties were reported by less affluent patients with locally recurrent disease (*p* = 0.003) and by less affluent survivors, i.e. patients cancer free for >2 years (*p* = 0.016) Wright 2015 -2688/17,830 (15.1%) were classified as socially distressed (summary score ≥ 10) -19.5% had difficulties with everyday living (score ≥ 5), 15.6% had difficulties with money matters (score ≥ 2) and 18.1% had difficulties with self and others (score ≥ 3) -Multiple regression analysis showed that the strongest predictor for social distress was having ≥3 long-term co-morbidities, followed by unemployment, recurrent or non-treatable disease and having a permanent stoma -Additional predictors of social difficulties were younger age (<55 years), living in a more deprived area, non-white ethnicity, tumour site, having advanced disease, having had surgery, radiotherapy or chemotherapy and being a carer (all *p*s < 0.001)

*s.d.* standard deviation

Most articles comprised single time-point surveys covering a broad array of tumour sites. The majority examined mixed tumour groups, but 7/22 reported on single disease groups. Sample size varied widely, ranging from 26 to over 21,000 patients. Overall, sex representation was clearly specified for all of the studies, but details of participants’ ages were less consistently provided. Most of the studies had been conducted within a time frame of the first 3 years following diagnosis. Some study samples were early disease, some clearly advanced disease and others a mixture or details were not provided within the article; however, no specification of stage was made in the inclusion criteria for the current review.

### Quality of publications

Marked against the quality criteria in Box 2, and reported in Table 1, only four studies scored highly (+++) [47, 57, 65, 66]. Some individual criteria warrant specific comment. Only 8 studies were rated as having bias free participant selection, 8 were rated as being apparently free of confounding factors and 11 accounted for all participants in their reporting. Of particular note was the use of diverse, non-validated, study-specific instruments to investigate financial distress; only two of 10 studies used a validated measure.

### Financial distress

Table 2 details the 10 articles [45–54] that reported on self-perceived financial distress. Only two used a validated measure. One [47] employed ‘The COST measure’ [68], a recently developed 11-item tool validated in patients with advanced cancer and available via the Functional Assessment of Chronic Illness Therapy (FACIT) suite of questionnaires (see www.facit.org). The second [48] used the ‘Personal Financial Wellness (PFW) Scale’ [69] **(**formally known as the ‘Incharge Financial Distress/Financial Well-Being Scale’ [70]), which has 8 items, is validated and has been used with cancer patients but without normative data [71]. The remaining eight studies [45, 46, 49–54] used un-validated study-specific tools. The number of questions used in these non-validated measures varied widely, and the content of the items across the measures was diverse and highly individual. Outcomes from the 10 articles show the presence of much self-perceived financial distress amongst the study participants. The findings also demonstrated strategies frequently employed by patients to mitigate their financial burden, including financial reductions to health-related areas and more generally with clothing, food and leisure. Additionally, patients borrowed money, used credit and defaulted on bills. Factors repeatedly associated with greater financial burden were younger age, lower income, poorer health status, more treatment/s, non-white ethnicity and poorer psychological well-being (i.e. greater anxiety and depression).

### Roles and responsibilities

Three articles [55–57] investigated the impact on patients’ roles and responsibilities in everyday life. Two [55, 56] used the ‘Parenting Concerns Questionnaire’ (PCQ) [72]. Park and colleagues [56] found that patients had mild to moderate concerns about their cancer affecting their parental role, this was both in terms of performing the usual practical things parents do for their children, but was also about the emotional consequences for the child. Of particular concern was how the child would cope with their death, impact of cancer on the child’s emotions and impact on the co-parent’s emotions. Factors associated with higher levels of concerns about parenting were single marital status, poor functional status, poorer quality of life, psychological distress (anxiety and depression) and lack of social support.

Moore et al. [55] combined the use of the PCQ with another tool, the ‘Parental Efficacy Beliefs Scale’, a non-validated instrument measuring patients’ perceptions of their own ability, and that of their co-parent to perform the parental role. Most (60%) felt they had been extremely well able to perform their parenting responsibilities before diagnosis, dropping to 11% after. Similarly, patients reported less belief in the co-parent after diagnosis (42% dropping to 26%). Factors associated with reported decline in parental efficacy were more frequent clinic visits, poorer quality of life and depression. Higher levels of parenting concerns expressed on the PCQ were accompanied by decline in patients’ belief about themselves and their co-parent being able to parent.

Walsh et al. [57] investigated the impact for women with breast cancer on their roles as wife and mother using the ‘Marital’ and ‘Relationship with Children’ subscales from the CARES instrument [73]. Talking to their spouse/partner about what may happen after their death was problematic for 52.8% of the women, and 37.4% indicated that just talking about cancer with them was difficult. Levels of reported difficulty on the Relationship with Children subscale were not high; however, 19.7% reported problems helping their children talk about their cancer, 16.8% helping their children cope and 12.7% taking care of their children as a result of breast cancer.

### Multiple aspects of social life

Nine articles used multidimensional instruments [58–66, N.B. 59 and 60 used the same data set]. Five [58–60, 62, 64] utilised the illness intrusiveness rating scale (IIRS) [74], a validated tool which measures the extent to which an illness and/or treatment affects the patient’s social life. Greater levels of intrusiveness were found to be associated with advanced disease, younger age, lower income, cancer type, recent treatment and poor psychological well-being (depression and hopelessness). Conversely, being older, not having children under 18 years living at home, having early stage disease, fewer symptoms and better psychosocial well-being were all associated with consistently low levels of illness intrusiveness. [64].

The finding that cancer and its treatments commonly impact the social aspects of patients’ lives was evident in three papers [63, 65, 66] that had used the Social Difficulties Inventory (SDI), a validated and psychometrically tested instrument [10, 75–78]. In one article with pooled data from three studies, 45% of participants reported high levels of social difficulty, which was associated more with younger age, those with advanced disease and, to some extent, those from deprived areas [65]. In another study, greater social difficulties were found to be associated with invasive disease (lymph node involvement or distant metastases), undergoing surgery, receiving chemotherapy and lower socioeconomic status, and a reduction in difficulties occurred by 10 months follow-up with the apparent disparity between low and high socioeconomic status groups disappearing [63]. Results from nearly 18,000 patients endorsed previous reports that predictors of social difficulties include younger age, living in a more deprived area, having advanced disease and undergoing surgery or chemotherapy [66]. This study also found additional predictive factors were tumour site, non-white ethnicity, receipt of radiotherapy and being a caregiver. The strongest predictor for social distress was found to be having ≥3 long-term co-morbidities, followed by unemployment and recurrent or non-treatable disease.

Social and financial impacts were also reported using a non-validated questionnaire in a study contrasting rural and urban cancer patients’ experiences [61]. Sixty-four percent of patients experienced at least one financial or social consequence, most frequently this was time-off work (44%), reduced income (31%) and missing family events (23%). City dwellers reported significantly more financial impact, but no similar difference was evident for social impact. Those who were younger, currently working and suffering stress, were found to be at higher risk of experiencing financial or social impacts.

## Discussion

Though the studies reviewed here were diverse in terms of study objectives, methods and patient populations, the patient-reported outcomes reported by them repeatedly show cancer and its treatments have significant impact on patients’ finances, roles and responsibilities and on the various facets of their social life.

Moreover, the findings complement reports from published quantitative studies [26–33] that have asked patients to report impact objectively (e.g. by reporting how much money they have spent on an item such as a medication, by indicating the time point of return to work or by indicating ability to perform work tasks) and qualitative studies [34–40] providing first-hand patient accounts of burden. All of this together underlines the far broader nature of the aspects of patients’ social lives that are impacted than can be assessed by relying solely on a social function subscale from one of the validated HRQoL instruments frequently in use, e.g. EORCT-QLQ-C30 [6] or FACT-G [7].

The studies reviewed which have attempted to quantify patients’ self-perceived financial distress have all done so using different, and in the majority of cases [45, 46, 49–54], study-specific measures. This has led to diverse and highly individualised content all scored with incomparable rating scales. This makes summarising findings across studies problematic. However, interest and concern regarding financial burden for patients with cancer have been growing recently [24], driven by the rapid pace of change where expensive novel drugs and living longer with incurable disease are now integral to the picture. In response to this pressure, at least one validated PRO, the COST measure [68], has been developed. To improve the quality of studies conducted and data collected in the future, validated instruments such as this need to be consistently and widely used. This may help researchers and healthcare professionals to better explore, understand and help ameliorate financial distress that patients may experience as a consequence of their cancer and treatments. The current review highlights a paucity of work regarding PROs used to quantify the impacts on patients’ everyday roles and responsibilities in life as a result of cancer and its treatments. Only three studies [55–57] were found, each exploring the impact on the parental role with validated tools. However, data are predominantly from female participants (see Table 1) which should be addressed with future work. The impact on any role/responsibility a patient might have towards other dependants such as elderly parents or an incapacitated spouse/partner was covered with only a single generic item on the SDI, ‘Have you had difficulty with looking after those who depend on you? (e.g. children, dependent adults, pets)’. Yet, evidence suggests that these aspects need to be included in our evaluations; exploratory qualitative analysis has shown that older women with breast cancer who cared for others struggled to balance caregiving responsibilities and their own health needs [36]. In another study, women with early-stage breast cancer and multiple caregiving roles were found to have elevated depressed mood over time [39].

Additionally, there is a survey literature [26–33], not reviewed here, documenting return to work rates and the assessment of work-related functional abilities measured using instruments such as the ‘Work Ability Index’ [79] or the ‘Work Limitations Questionnaire’ [80] in patients with, or treated for, cancer. A few qualitative studies [34, 35, 40] have described the value and meaning of paid work in patients’ lives and barriers to re-joining the workforce. However, the current review found little of patients’ own perceptions of the impact of cancer and its treatments on these aspects of life measured using a comprehensive PRO. The two validated multidimensional instruments identified (IIRS, SDI) have single items asking about impact on work amounting to nothing above that already found in the frequently used, validated, HRQoL instruments (EORCT-QLQ-C30 or FACT-G).

Two validated multidimensional PROs were identified, the IIRS [74] and SDI [10], which capture a broad range of aspects of patients’ everyday lives albeit balanced with brevity. The SDI is of particular relevance since it has been specifically developed for the oncology setting with the aim of being used as a tool to facilitate the integration of the assessment of social well-being into routine care in a way similar to that advocated for psychological well-being [76, 77]. Its purposes are to aid detection and characterisation of social problems and to improve communication between patients and healthcare professionals about this, leading to appropriate service responses, including if necessary onward referral and ultimately better care and enhanced well-being for patients. Other ways that it, and similar instruments, could be used include for the research and development of interventions to help patients manage social impacts like financial burden or family caregiving responsibilities, or perhaps as part of survivorship care plans, as well as for assessing any differential impacts of treatments being compared in clinical trials.

Nevertheless, clearly insufficient data are being gathered with patients to comprehensively understand their problems and needs to enable supporting them living well with their disease, including making decisions about ongoing treatment options. Impacts on the social domain need to be consistently assessed more comprehensively and this has implications for the current HRQoL instruments commonly used. Options for the future include using the SDI in tandem with the existing well-validated quality of life measures and/or generating further assessment modules (i.e. subscales) for the existing instruments.

## Limitations

This review was intentionally restricted to identifying studies that reported the use of PRO instruments dedicated solely to the assessment of the financial situation, lifestyle, occupational circumstances, and roles and responsibilities with dependents (looking after young offspring or elderly parents) of adult cancer patients living with advanced disease. In taking this approach, we acknowledge that we have not conducted a mapping and review process of existing social domain subscales of either the general or the more specific health-related quality of life instruments available to use in oncology and that such an undertaking could reveal further useful validated measurement items. One such tool absent from the current review is the Patient-Reported Outcomes Measurement Information System (PROMIS) [81], a product of the National Institutes of Health (NIH) in the USA. This is because existing literature on its social health domain measure (which covers companionship, social isolation, social roles and activities and social support) [82] is about its development and psychometrics, meaning it either did not meet the search terms and/or inclusion criteria or did meet an exclusion criterion. However, it is likely to have significant presence in the future, and an awareness of it is important. It has an item bank approach to its method of development, has been evolved with patients and content experts and has undergone rigorous testing and validation continues to be ongoing. It has a scoring system to norm responses to the general US population with free access and use.

## Conclusions

There is a need and opportunity for further work, both in terms of instrument use and development, to ensure that the social areas of patients’ lives are comprehensively assessed. Our tools must keep pace with changes happening in cancer treatment outcomes so that salient social problems are anticipated and/or ameliorated. The COST and SDI, two well-developed and validated tools, could be used to supplement shortfalls in the commonly employed HRQoL instruments. But, there is also scope for developing additional subscales within the existing modular systems to enhance them. In particular, attention needs to be directed towards roles and responsibilities, including work and patients with dependents.
